# Increasing adoption rates at animal shelters: a two-phase approach to predict length of stay and optimal shelter allocation

**DOI:** 10.1186/s12917-020-02728-2

**Published:** 2021-02-05

**Authors:** Janae Bradley, Suchithra Rajendran

**Affiliations:** 1 Department of Bioengineering, University of Missouri Columbia, Columbia, MO 65211 USA; 2 Department of Industrial and Manufacturing Systems Engineering, University of Missouri Columbia, Columbia, MO 65211 USA; 3 Department of Marketing, University of Missouri Columbia, Columbia, MO 65211 USA

**Keywords:** Animal shelter, High euthanization rates, Machine learning algorithms, Prediction models, Goal programming approach, Decision support tool

## Abstract

**Background:**

Among the 6–8 million animals that enter the rescue shelters every year, nearly 3–4 million (i.e., 50% of the incoming animals) are euthanized, and 10–25% of them are put to death specifically because of shelter overcrowding each year. The overall goal of this study is to increase the adoption rates at animal shelters. This involves predicting the length of stay of each animal at shelters considering key features such as animal type (dog, cat, etc.), age, gender, breed, animal size, and shelter location.

**Results:**

Logistic regression, artificial neural network, gradient boosting, and the random forest algorithms were used to develop models to predict the length of stay. The performance of these models was determined using three performance metrics: precision, recall, and F1 score. The results demonstrated that the gradient boosting algorithm performed the best overall, with the highest precision, recall, and F1 score. Upon further observation of the results, it was found that age for dogs (puppy, super senior), multicolor, and large and small size were important predictor variables.

**Conclusion:**

The findings from this study can be utilized to predict and minimize the animal length of stay in a shelter and euthanization. Future studies involve determining which shelter location will most likely lead to the adoption of that animal. The proposed two-phased tool can be used by rescue shelters to achieve the best compromise solution by making a tradeoff between the adoption speed and relocation cost.

## Background

As the problem of overpopulation of domestic animals continues to rise, animal shelters across the nation are faced with the challenge of finding solutions to increase the adoption rates. In the United States, about 6–8 million dogs and cats enter animal shelters every year, and 3–4 million of those animals are euthanized [1]. In other words, about 50% of the total canines and felines that enter animal shelters are put to death annually. Moreover, 10–25% of the total euthanized population in the United States is explicitly euthanized because of shelter overcrowding each year [2]. Though animal shelters provide incentives such as reduced adoption fees and sterilizing animals before adoption, only a quarter of total animals living in the shelter are adopted.

### Animal adoption from shelters and rescues

There are various places to adopt an animal, and each potential owner must complete the adoption process and paperwork to take their new animal home [3]. Public and private animal shelters include animal control, city and county animal shelters, and police and health departments. Staff and volunteers run these facilities. Animals may also be adopted from a rescue organization, where pets are fostered in a home or a private boarding facility. These organizations are usually run by volunteers, and animals are viewed during local adoption events that are held at different locations, such as a pet store [3].

There could be several reasons for the euthanization of animals in a shelter, such as overcrowding, medical issues (ex. sick, disabled), or behavioral issues (ex. too aggressive). The causes for the overpopulation of animals include failure to spay or neuter animals leading to reckless breeding habits and abandonment or surrender of offspring, animal abandonment from owners who are no longer able to take care of or do not want the animal, and individuals still buying from pet stores [4]. With the finite room capacity for animals that are abandoned or surrendered, overpopulation becomes a key challenge [5]. Though medical and behavioral issues are harder to solve, the overpopulation of healthy adoptable animals in shelters is a problem that can be addressed through machine learning and predictive analytics.

## Literature review

In this section, we describe the research conducted on animal shelters evaluating euthanasia and factors associated with animal adoption. The articles provide insights into factors that influence the length of stay and what characteristics influence adoption.

Studies have been conducted investigating the positive influence of pre-adoption neutering of animals on the probability of pet adoption [2]. The author investigated the impact of the cooperation of veterinary medical schools in increasing pet adoption by offering free sterilization. Results demonstrated that the collaboration between veterinary hospitals and local animal shelters decreased the euthanization of adoptable pets.

Hennessy et al. [6] conducted a study to determine the relationship between the behavior and cortisol levels of dogs in animal shelters and examined its effect in predicting behavioral issues after adoption. Shore et al. [7] analyzed the reasons for returning adopted animals by owners and obtained insights for these failed adoptions to attain more successful future approvals. The researchers found that prior failed adoption had led to longer-lasting future acceptances. They hypothesized that the failed adoptions might lead owners to discover their dog preferences by assessing their living situation and the type of animal that would meet that requirement.

Morris et al. [8] evaluated the trends in income and outcome data for shelters from 1989 to 2010 in a large U.S. metropolitan area. The results showed a decrease in euthanasia, adoption, and intake for dogs. For cats, a reduction in intake was observed until 1998, a decrease in euthanasia was observed until 2000, and the adoption of cats remained the same. Fantuzzi et al. [9] explored the factors that are significant for the adoption of cats in the animal shelter. The study investigated the effects of toy allocation, cage location, and cat characteristics (such as age, gender, color, and activity level). Results demonstrated that the more active cats that possessed toys and were viewed at eye level were more likely to impress the potential adopter and be adopted. Brown et al. [10] conducted a study evaluating the influence of age, breed, color, and coat pattern on the length of stay for cats in a no-kill shelter. The authors concluded that while color did not influence the length of stay for kittens, whereas gender, coat patterning, and breed were significant predictors for both cats and kittens.

### Machine learning

Machine learning is one possible tool that can be used to identify risk factors for animal adoption and predict the length of stay for animals in shelters. Machine learning is the ability to program computers to learn and improve all by itself using training experience [11]. The goal of machine learning is to develop a system to analyze big data, quickly deliver accurate and repeatable results, and to adapt to new data independently. A system can be trained to make accurate predictions by learning from examples of desired input-output data. More specifically, machine learning algorithms are utilized to detect classification and prediction patterns from large data and to develop models to predict future outcomes [12]. These patterns show the relationship between the attribute variables (input) and target variables (output) [13].

**Fig. 1:**
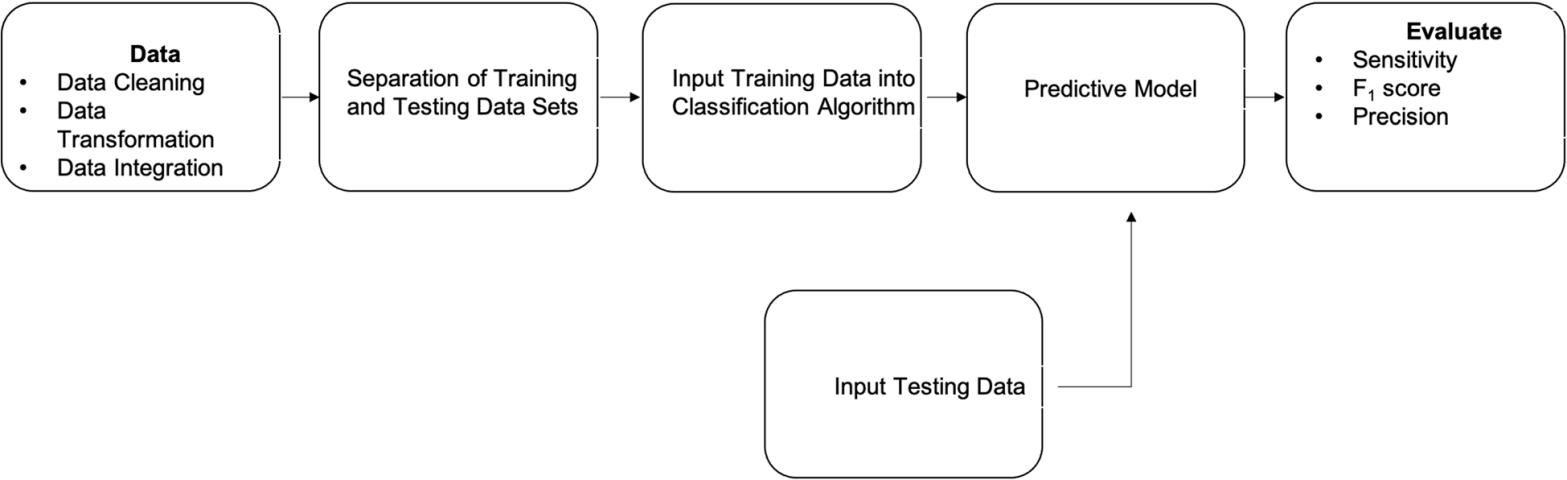
Pictorial Representation of Developing a Predictive Model

Widely used data mining tasks include supervised learning, unsupervised learning, and reinforcement learning [14]. Unsupervised learning involves the use of unlabeled datasets to train a system for finding hidden patterns within the data [15]. Clustering is an example of unsupervised learning. Reinforcement learning is where a system is trained through direct interaction with the environment by trial and error [15]. Supervised learning encompasses classification and prediction using labeled datasets [15]. These classification and regression algorithms are used to classify the output variable with a discrete label or predict the outcome as a continuous or numerical value. Traditional algorithms such as neural networks, decision trees, and logistic regression typically use supervised learning. Figure 1 provides a pictorial of the steps for developing and testing a predictive model.

### Contributions to the literature

Although prior studies have investigated the impact of several factors, such as age and gender, on the length of stay, they focus on a single shelter, rather than multiple organizations, as in this study. The goal of this study is to investigate the length of stay of animals at shelters and the factors influencing the rate of animal adoption. The overall goal is to increase adoption rates of pets in animal shelters by utilizing several factors to predict the length of stay. Machine learning algorithms are used to predict the length of stay of each animal based on numerous factors (such as breed, size, and color). We address several objectives in this study that are listed below.

1. Identify risk factors associated with adoption rate and length of stay
2. Utilize the identified risk factors from collected data to develop predictive models
3. Compare statistical models to determine the best model for length of stay prediction

## Results

### Exploratory Data results

**Fig. 2:**
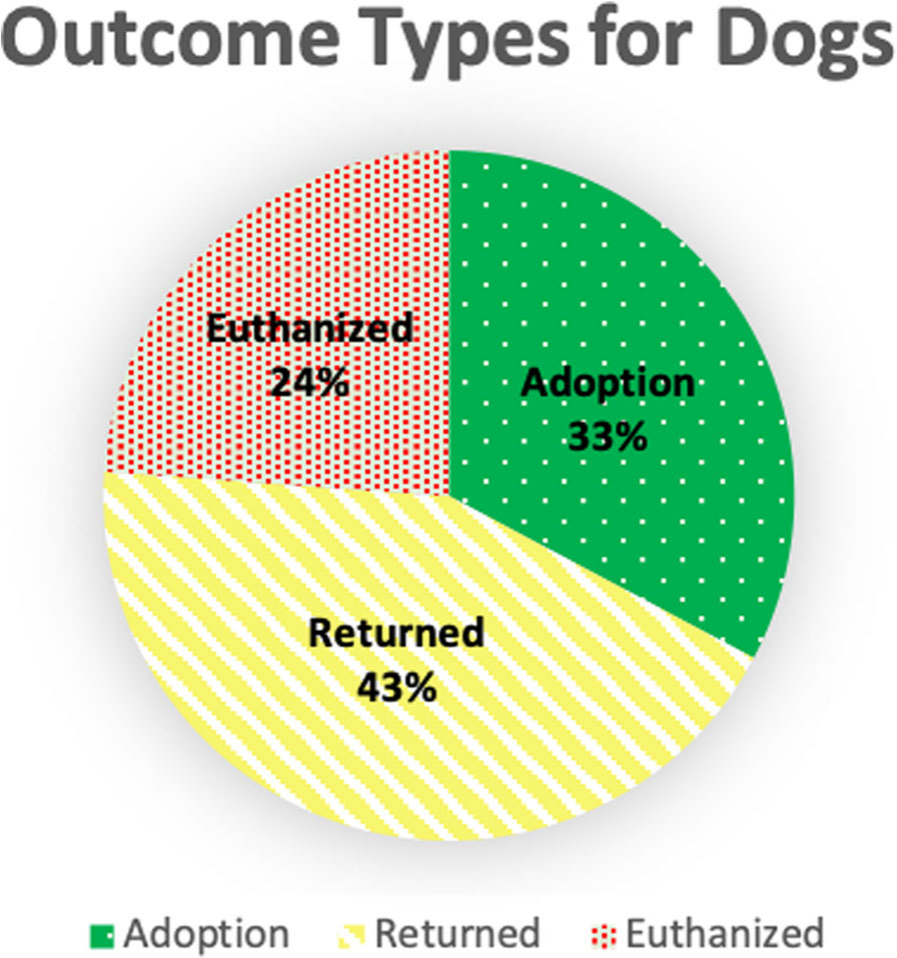
Distribution of Outcome Types for Dogs

**Fig. 3:**
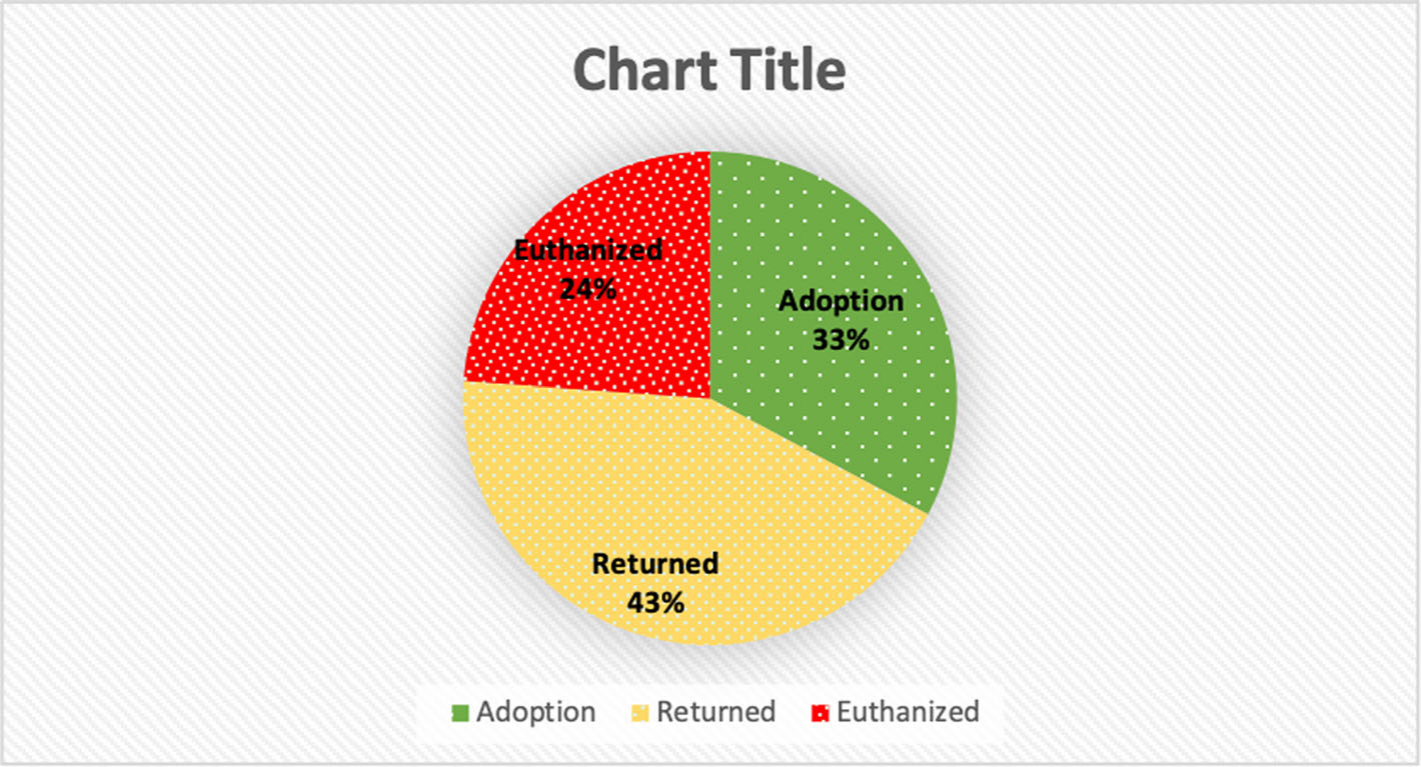
Distribution of Outcome Types for Cats

From Fig. 2, it is evident that the return of dogs is the highest outcome type at 43.3%, while Fig. 3 shows that the adoption of cats is the highest outcome type at 46.1%. Both figures illustrate that the euthanization of both cats and dogs is still prevalent (~ 20%). The results from Table 1 demonstrate that the longest time spent in the shelter is at 355 days by a male cat that is adopted and a female dog that is euthanized. Observing the results, adoption has the lowest variance among all animal types compared to the other outcome types. Adopted male cats have the lowest variance for days spent in the shelter, followed by female dogs. Female cats that are returned have the highest variance for days spent in the shelter.

**Table 1:** Data Summary

Type	Adopt			Return			Euthanize		
	Mean	St. Dev	CV	Mean	St. Dev	CV	Mean	St. Dev	CV
Male Dogs	21.87	24.74	1.13	8.52	15.74	1.85	10.69	20.75	1.94
Female Dogs	21.17	23.65	1.12	8.56	15.31	1.79	9.64	20.00	2.07
Male Cats	37.75	40.78	1.08	6.32	13.69	2.17	6.59	11.73	1.78
Female Cats	29.68	33.63	1.13	7.53	16.83	2.24	7.15	11.02	1.54

**Fig. 4:**
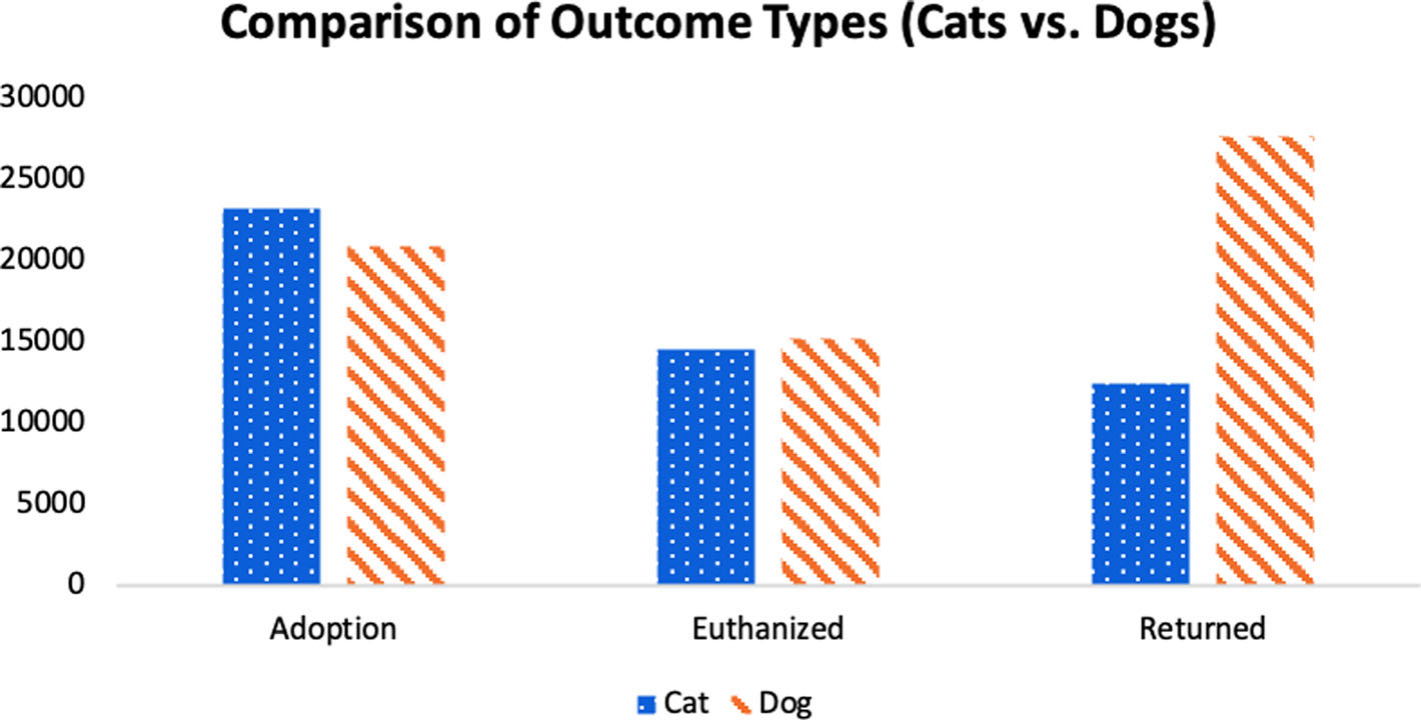
Comparison of Outcome Types for Cats and Dogs

**Fig. 5:**
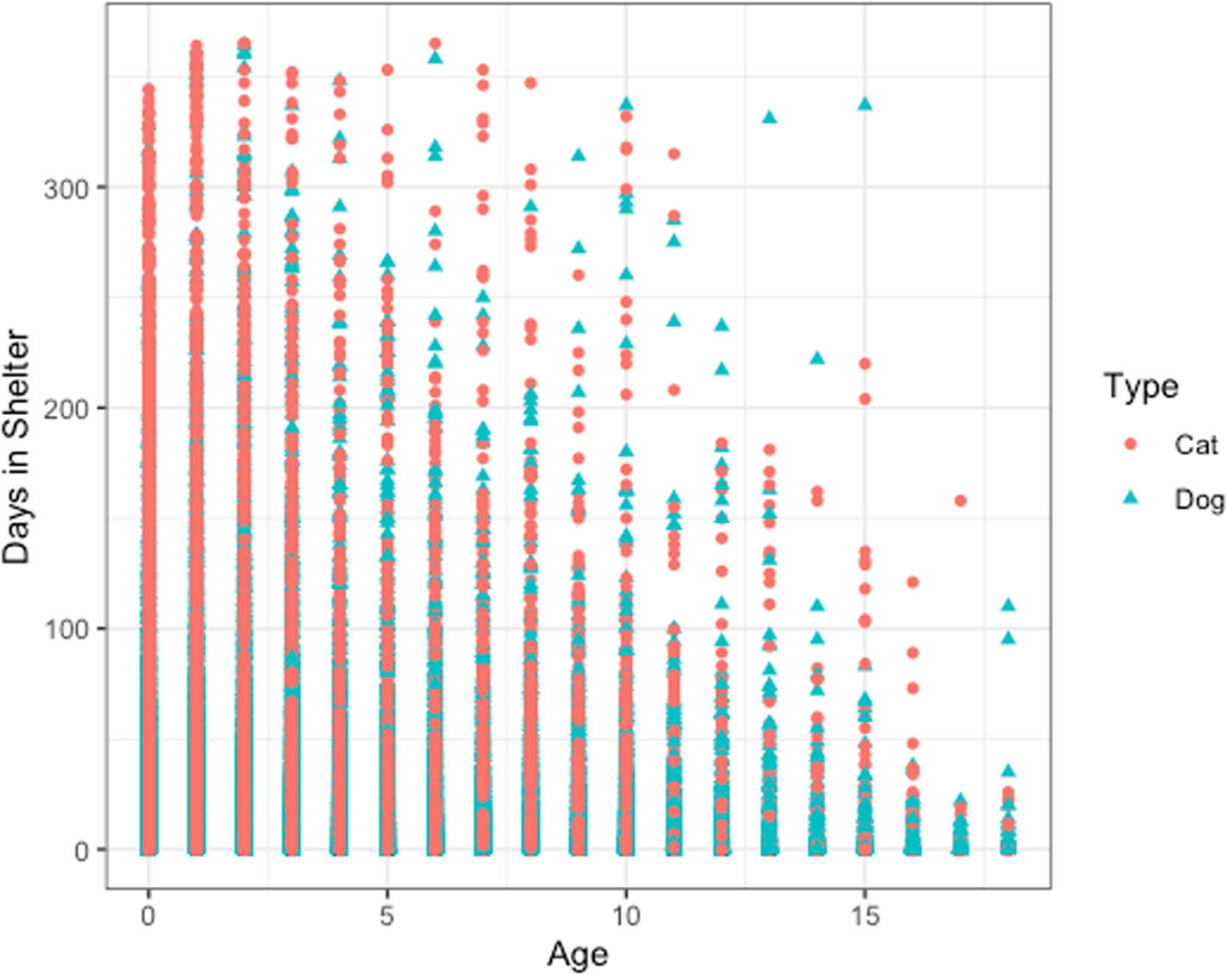
Age vs. Days in Shelter for Cats and Dogs

Figure 4 shows a comparison of cats and dogs for the three different outcome types. It is observed from the data that there are more dogs returned than cats. From Fig. 5, it is observed that the number of days a dog stays in the shelter decreases as the age increases. This is not expected, as it is predicted that the number of days in a shelter would be lower for younger dogs and puppies. This observation could be due to having more data points for younger dogs.

### Machine learning results

Examining Table 2, it is clear that the most proficient predictive model is developed by the gradient boosting algorithm for this dataset, followed by the random forest algorithm. The logistic regression algorithm appears to perform the worst with low precision, recall, and F1 score performance metrics for all categories of length of stay. For the prediction of low length of stay in a shelter, the random forest algorithm is the best performing model in comparison to the others at around 64–70% performance for precision, recall, and F1 score. The ANN algorithm is found to be the best when evaluating the precision and F1 score for medium length of stay, while the random forest algorithm is better for assessing recall. However, the performance of these models in predicting the medium length of stay for the given dataset is low for all three-performance metrics. The gradient boosting algorithm performs the best when predicting the high length of stay. Finally, the gradient boosting and random forest algorithms perform well when predicting the very high length of stay at around 70–80%.

**Table 2:** Consolidated Results

Length of Stay	Precision	LR			ANN			RF			GB	
		Recall	F1 Score	Precision	Recall	F1 Score	Precision	Recall	F1 Score	Precision	Recall	F1 Score
Low	0.4171	0.3852	0.4005	0.5508	0.513	0.5312	0.6441	0.703	0.6723	0.6264	0.4631	0.5325
Medium	0.386	0.3716	0.3786	0.4181	0.5146	0.4614	0.3068	0.5533	0.3948	0.2254	0.5043	0.3116
High	0.3072	0.4145	0.3529	0.4155	0.2707	0.3278	0.3832	0.6197	0.4736	0.7251	0.7188	0.7219
Very High (Euthanization)	0.4536	0.4213	0.4369	0.5612	0.5209	0.5403	0.7031	0.7138	0.7084	0.7902	0.7096	0.7477
Average	0.391	0.3981	0.3922	0.4864	0.4548	0.4652	0.5093	0.6474	0.5623	0.5918	0.5989	0.5784
Computational Time (s)		9.41			2023			2139			2009	

Results from Table 2 also demonstrate that the model developed from the gradient boosting algorithm has a higher performance when predicting the high length of stay that leads to adoption, and when the outcome is euthanization. Evaluating the average of all three-performance metrics for all algorithms, the gradient boosting is the most proficient model at almost 60%, while logistic regression appears to be the worst. Table 2 also provides the computational time for each machine learning algorithm. For the given dataset, logistic regression runs the fastest at 9.41 s, followed by gradient boosting, artificial neural network, and finally, random forest running the longest. The gap in the performance measure (*pm*) is calculated by $\frac{p{m}_{best}-p{m}_{worst}}{p{m}_{best}}$, and is nearly 34, 39, and 32% for precision, recall, and F1 score, respectively.

Table 3 provides information on the top features or factors from each machine learning algorithm. Observing the table, we find that age (senior, super senior, and puppy), size (large and small), and color (multicolor) has a significant impact or influence on the length of stay. Specifically, we observe that older-aged animals (senior and/or super senior) appear as a significant factor for every algorithm. For the artificial neural network, older age is the #2 and #3 predictor, and super senior is the #2 predictor for the gradient boosting algorithm. Large and small-sized animals are also observed to be important features, as both are shown as the #1 predictor in the gradient boosting and ANN algorithms. The results also demonstrate that gender, animal type, other colors besides multicolor, middle age, and medium-sized animals did not significantly impact the length of stay.

**Table 3:** Top Three Features using Different Machine Learning Algorithms

Algorithm	Top Three Features		
	#1	#2	#3
**Logistic Regression**	Multicolor	Small Size	Senior
**ANN**	Large Size	Super Senior	Senior
**RF**	Puppy	Multicolor	Super Senior
**GB**	Small Size	Super Senior	Large Size

## Discussion

Results from our study provided information on what factors are significant in influencing length of stay. Brown et al. [10] conducted research that found that age, breed designation, coat color, and coat pattern influenced the length of stay for cats in animal shelters. Similar to these studies, observations from our study also suggest that age and color have a significant impact or influence on the length of stay.

Determining which algorithm will develop the best model for the given set of data is critical to predict the length of stay and minimize the chances of euthanization. The goal of predictive analytics is to develop a model that best approximates the true mapping function for the relationship between the input and output variables. To approximate this function, parametric or non-parametric algorithms can be used. Parametric algorithms simplify the unknown function to a known form. Non-parametric algorithms do not make assumptions about the structure of the mapping function, allowing free learning of any functional form. In this study, we utilize both parametric (logistic regression and artificial neural network) and non-parametric (random forest and gradient boosting) algorithms on the given data. Observing the results from Table 2, the gradient boosting and random forest (non-parametric algorithms) perform the best on the dataset. It is observed from the results that using a non-parametric approach leads to a better approximation of the true mapping function for the given records. These results also support prior studies on parametric versus non-parametric methods. Neely et al. [16] detailed the theoretical superiority of non-parametric algorithms for detecting pharmacokinetic and pharmacodynamic subgroups in a study population. The author suggests this superiority comes from the lack of assumptions made about the distribution of parameter values in a dataset. Bissantz et al. [17] discussed a resampling algorithm that evaluates the deviations between parametric and non-parametric methods to be noise or systematic by comparing parametric models to a non-parametric “supermodel”. Results demonstrate the non-parametric model to be significantly better. The use of algorithms that do not approximate the true function of the relationship between input and output provides better performance results for this application as well.

Current literature also supports the use of ensemble methods to increase prediction accuracy and performance. Dietterich [18] discussed the ongoing research into developing good ensemble methods as well as the discovery that ensemble algorithms are often more accurate than individual algorithms that are used to create them. Pandey, and S, T [19]. conducted a study to compare the accuracy of ensemble methodology on predicting student academic performance as research has demonstrated better results for composite models over a single model. This study applied ensemble techniques on learning algorithms (AdaBoost, Random Forest, Rotation Forest, and Bagging). For our study with the given records, the results support this claim. Both the gradient boosting and random forest algorithms are ensemble algorithms and performed the best on the animal shelter data.

Results from Table 2 demonstrate the best performance of the gradient boosting and random forest algorithm when the length of stay was classified as very high or the animal was euthanized. This is beneficial as the models can predict long stays where the outcome is euthanasia. This can lead to shelters identifying at-risk animals and implementing methods and solutions to ensure their adoption. These potential methods are the second phase of this research study, which will involve relocating animals to shelters where they will more likely be adopted. This phase is discussed in the future directions section.

Studies have been conducted evaluating euthanasia-related stress on workers (e.g., [1]). In other words, overpopulation not only leads to euthanasia but can, in turn, cause mental and emotional problems for the workers. For instance, Reeve et al. [20] evaluated the strain related to euthanasia among animal workers. Results demonstrated that euthanasia related strain was prevalent, and an increase in substance abuse, job stress, work causing family conflict, complaints, and low job satisfaction was observed. Predicting the length of stay for animals will aid in them being more likely to be adopted and will lead to fewer animals being euthanized, adding value not only to animals finding a home but also less stress on the workers.

The approach developed in this paper could be beneficial not only to reduce euthanasia but also to reduce overcrowding in shelters operated in countries where euthanasia of healthy animals is illegal, and all animals must be housed in shelters until adoption (or natural death). It is essential to develop an information system for a collaborative animal shelter network in which the entities can coordinate with each other, exchanging information about the animal inventory. Another benefit of this study is that it investigates applying machine learning to the animal care domain. Previous studies have looked into what factors influence the length of stay; however, this study utilizes these factors in addition to classification algorithms to predict how long an animal will stay in the shelter. Moreover, the use of a prescriptive analytics approach is discussed in this paper, where the predictions made by the machine learning algorithms will be used along with a goal programming model to decide in what shelter is an animal most likely to be adopted.

Limitations of this study include lack of behavioral data, limited sample size, and the use of simple algorithms. The first limitation, lack of behavioral data of the animal during intake and outcome, would be beneficial to develop a more comprehensive model. Though behavioral problems are harder to solve, having data would provide insight into how long these animals with behavioral issues are staying in shelters and what the outcome is. Studies have shown that behavioral problems play a significant role in preventing bonding between owners and their animals and one of the most common reasons cited for animal surrender [21, 22]. These behavioral problems can include poor manners, too much energy, aggression, and destruction of the household. Dogs surrendered to shelters because of behavioral issues have also been shown to be less likely to be adopted or rehomed, and the ones that are adopted are more likely to be returned [21]. Studies have also been conducted to evaluate the effect of the length of time on the behavior of dogs in rescue shelters [23–25]. Most of them concluded that environmental factors led to changes in the behavior of dogs and that a prolonged period in a shelter may lead to unattractive behavior of dogs to potential owners. Acquiring information on behavioral problems gives more information for the algorithm to learn when developing the predictive model. This allows more in-depth predictions to be made on how long an animal will stay in a shelter, which could also aid in adoption. This approach can be used to shorten the length of stay, which makes sure that healthy animals are not developing behavioral problems in the shelters. It is not only crucial for the animal to be adopted, but also that the adoption is a good fit between owner and pet. Shortening the length of stay would also lessen the chance that the animal will be returned by the adopter because of behavior. Having this information will also allow shelters to find other shelters close by where animals with behavioral issues are more likely to be adopted. To overcome this limitation of the lack of data on behavioral problems, behavioral issues will be used as a factor and will be specifically asked for when acquiring data from shelters.

Another limitation includes collecting more data from animal shelters across the United States, allowing for more representative data to be collected and inputted into these algorithms. However, this presents a challenge due to most shelters being underfunded and low on staff. Though we reached out to shelters, most replied that they lacked the resources and staff to provide the information needed. Future work would include applying for funding to provide a stipend to staff for their assistance in gathering the data from respective shelters. With more data, the algorithm has more information to learn on, which could improve the performance metrics of the predictive models developed. There may also be other factors that show to be significant as more data is collected.

Finally, the last limitation is the use of simpler algorithms. This study considers basic ML algorithms. Nevertheless, in recent years, there has been development in the ML field of more complex networks. For instance, Zhong et al. [26] proposed a novel reinforcement learning method to select neural blocks and develop deep learning networks. Results demonstrated high efficiency in comparison to most of the previous deep network search approaches. Though only four algorithms were considered, future work would investigate deep learning networks, as well as bagging algorithms. Using more complex algorithms could ensure that if intricate patterns in the data are present, the algorithm can learn them.

### Future direction

#### Phase 2: goal programming approach for making relocation decisions

Using the information gathered in this study, we can predict the type of animals that are being adopted the most in each region and during each season of the year. To accomplish this, we utilize a two-phase approach. The first phase was leveraging the machine learning algorithms to predict the length of stay of each animal based on numerous factors (such as breed, size, and color). Phase-2 involves determining the best shelter to transport adoptable animals to increase the adoption rates, based on several conflicting criteria. This criterion includes predicted length of stay from phase-1, the distance between where the animal is currently housed and the potential animal shelters, transportation costs, and transportation time. Therefore, our goal is to increase adoption rates of pets in animal shelters by utilizing several factors to predict the length of stay, as well as determine the optimal animal shelter location where the animal will have the least amount stay in a shelter and most likely be adopted.

After predicting the length of stay of an incoming animal that is currently housed in the shelter *l*^′^ using the machine learning algorithms, the next phase is to evaluate the potential relocation options for that animal. This strategic decision is specifically essential if the length of stay of the animal at its current location is high/very high. Nevertheless, while making this relocation decision, it is also necessary to consider the cost of transporting the animal between the shelters. For instance, if a dog is brought into a shelter in Houston, Texas, and is estimated to have a high/very high length of stay. Suppose if the dog is predicted to have a low length of stay at New York City and a medium length of stay at Oklahoma City, then a tradeoff has to be made between the relocation cost and the adoption speed. The objectives, length of stay, and relocation costs are conflicting and have to be minimized. Phase-2 attempts to yield a compromise solution that establishes a trade-off between these two criteria.

Goal programming (GP) is a widely used approach to solve problems involving multiple conflicting criteria. Under this method, each objective function is assigned as a goal, and a target value is specified for the individual criterion [27]. These target numbers can be fulfilled by the model with certain deviations, while the objective of the GP model is to minimize these deviations. Pertaining to this study, the desired values for the length of stay and relocation cost is pre-specified in the model and can be fulfilled with deviations. The GP model attempts to minimize these deviations. Thus, this technique attempts to produce a solution that is as close as possible to the targets, and the model solutions are referred to as the “most preferred solution” by prior studies (e.g., [28, 29]).

As mentioned earlier, the primary task to be completed using this phase-2 goal programming approach is the relocation decisions considering the adoption speed and the cost of transporting the animal from the current location.

#### Model notations

**Table:** 

Sets and Indices:	
*l* ∈ *L*	Set of shelter locations
Parameters:	
*l*^′^	Index for shelter location that is currently hosting the incoming animal
*s*	Size of the animal (small, medium, and large)
*y*	Type of animal (dog, cat, etc.)
*e*_*l*_	Length of stay of the animal at shelter *l*. *e*_*l*_ is categorical and is obtained from the output of Phase-1.
${r}_{l^{\prime },l}$	Relocation cost if the animal is transported between shelters *l*^′^ (that is currently hosting the animal) and *l*
*t*_*l*, *y*, *s*_	Remaining housing units available for animal type *y* of size *s* at shelter *l*. Typically shelters are designed such that there is a fixed number of rooms for each animal’s size and type.
*u*^*LS*^	Upper bound on the length of stay
*u*^*RC*^	Upper bound on the relocation cost
Goal Parameters:	
*p*^*s*^	Desired value for the preferred length of stay
*p*^*r*^	Desired value for the preferred relocation cost
Variables:	
*X*_*l*_	if the incoming animal is assigned to shelter *l*
	0 otherwise
${d}_g^{-}$	Negative deviation variable for goal *g*
${d}_g^{+}$	Positive deviation variable for goal *g*

#### Goal programming model formulation

##### Goal constraints

Objective 1: Minimize the overall length of stay of the animal under consideration (Eq. 1).

1 $$ \operatorname{Minimize}\kern0.50em {LS}^O={\sum}_{l=1}^L{e}_l{X}_l $$

Goal constraint for objective 1: The corresponding goal constraint of objective 2 is given using Equation [30].

2 $$ {\sum}_{l=1}^L{e}_l{X}_l+{d}_1^{-}-{d}_1^{+}={p}^s $$

Objective 2: Minimize the overall relocation cost for transporting the animal under consideration (Eq. 3).

3 $$ \operatorname{Minimize}\ {\mathrm{RC}}^O={\sum}_{\begin{array}{l}l=1\\ {}l\ne l\hbox{'}\end{array}}^L{r}_{l\hbox{'},l}{X}_l $$

Goal constraint for objective 2: The corresponding goal constraint of objective 2 is given using Equation [18].

4 $$ {\sum}_{\begin{array}{c}l=1\\ {}l\ne {l}^{\prime}\end{array}}^L{r}_{l^{\prime },l}{X}_l+{d}_2^{-}-{d}_2^{+}={p}^r $$

##### Hard constraints

Equation [9] ensures that the animal can be assigned to only one shelter.

5 $$ {\sum}_{l=1}^L{X}_l=1 $$

The animal can be accommodated in shelter *l* only if there are a shelter capacity and type for that particular animal size category, and this is guaranteed using constraint [31]. It is important to note that both *y* and *s* are input *parameters*, whereas *l* is the *set* of shelters.

6 $$ {X}_l\le {t}_{l,y,s}\kern2.4em \forall l $$

Equation [21] sets an upper limit on the length of stay category if the shelter *l* is assigned as the destination location. This prevents relocating animals to a shelter that might potentially have a high or very high length of stay.

7 $$ {e}_l\times {X}_l\le {u}^{LS}\kern2.52em \forall l $$

Similarly, Equation [32] sets an upper limit on the relocation cost, if the shelter *l* is assigned as the destination location. This prevents relocating animals to a very far location. The current shelter location, *l*^′^, that is hosting the animal is an input parameter.

8 $$ {r}_{l\hbox{'}l}\times {X}_l\le {u}^{RC}\kern2.4em \forall l $$

##### Objective function

Since the current problem focuses on minimizing the expected length of stay and relocation cost, the objective function of the goal programming approach is to reduce the sum of the weighted positive deviations given in Equations ([18, 30], as shown in Equation [6].

9 $$ \operatorname{Minimize}\ \mathrm{Z}={\mathrm{w}}_1{d_1}^{+}+{w}_2{d_2}^{+} $$

where *w*_*g*_ is the weight assigned for each goal *g*.

It is necessary to scale the deviation (since the objectives have different magnitudes as well as units) to avoid a biased solution.

If the scaling factors are represented by *f*_*g*_ for goal *g*, then the scaled objective function is given in Equation [14].

10 $$ \operatorname{Minimize}\ \mathrm{Z}=\frac{w_1\times {d}_1^{+}}{f_1}+\frac{w_2\times {d}_2^{+}}{f_2} $$

Using this goal programming approach, the potential relocation options are evaluated considering the length of stay from phase-1. This phase-2 goal programming approach is useful, especially if the length of stay of the animal at its current location is high/very high, and a trade-off has to be made between relocation cost and length of stay. Phase-2 acts as a recommendation tool for assisting administrators with relocation decisions.

## Conclusion

Nearly 3–4 million animals are euthanized out of the 6–8 million animals that enter shelters annually. The overall objective of this study is to increase the adoption rates of animals entering shelters by using key factors found in the literature to predict the length of stay. The second phase determines the best shelter location to transport animals using the goal programming approach to make relocation decisions. To accomplish this objective, first, the data is acquired from online sources as well as from numerous shelters across the United States. Once the data is acquired and cleaned, predictive models are developed using logistic regression, artificial neural network, gradient boosting, and random forest. The performance of these models is determined using three performance metrics: precision, recall, and F1 score.

The results demonstrate that the gradient boosting algorithm performed the best overall, with the highest precision, recall, and F1 score. Followed closely in second is the random forest algorithm, then the artificial neural network, and then finally, the logistic regression algorithm is the worst performer. We also observed from the data that the gradient boosting performed better when predicting the high or very high length of stay. Further observing the results, it is found that age for dogs (e.g., puppy, super senior), multicolor, and large and small size are important predictor variables.

The findings from this study can be utilized to predict how long an animal will stay in a shelter, as well as minimize their length of stay and chance of euthanization by determining which shelter location will most likely lead to the adoption of that animal. For future studies, we will implement phase 2, which will determine the best shelter location to transport animals using the goal programming approach to make relocation decisions.

## Methods

### Data description

A literature review is conducted to determine the factors that might potentially influence the length of stay for animals in shelters. These factors include gender, breed, age, and several other variables that are listed in Table 4**.** These features will be treated as input variables for the machine learning algorithms. Overall, there are eight input or predictor variables and one output variable, which is the length of stay.

**Table 4:** Factor Description

Variable	Description	Variable Type
Type	Cat or Dog	Categorical
Breed	Breed of the animal (e.g., labrador retriever, beagle)	Categorical
Color	Color of the animal (e.g., black, brown, white, multi-colored)	Categorical
Gender	Gender of the animal (male/female)	Categorical
Age	Age of the animal categorized as puppy/kitten, adult, senior, and super senior	Categorical
Location	Shelter location where dog or cat is housed	Categorical
Outcome Type	End outcome after the animal is brought into the shelter (euthanized, adopted, and returned)	Categorical
Length of Stay	The time that the animal spends in shelter categorized as low, medium, high, and very high (i.e., euthanized)	Categorical

Animal shelter intake and outcome data are publicly made available by several state/city governments on their website (e.g., [33, 34]), specifically in several southern and south-western states. These online sources provide datasets for animal shelters from Kentucky (150,843 data rows), California (334,016), Texas (155,115), and Indiana (4132). Since there is no nationwide database for animal shelters, information is also collected through individual animal shelters that conduct euthanization of animals. We contacted over 100 animal shelters across the United States and inquired for data on the factors mentioned in Table 4. We received responses from 20 of the animal shelters that were contacted. Most responses received stated there was not enough staff or resources to be able to provide this information. From the responses that were received back, only four shelters were able to provide any information. Of those four, only two of the datasets contained the factors and information needed, which are Colorado (8488 data rows) and Arizona (4, 667 data rows).

The data that is collected from the database and animal shelters included information such as animal type, intake and outcome date, gender, color, breed, and intake and outcome status (behavior of animal entering the shelter and behavior of animal at outcome type). These records also included information on several types of animals, such as dogs, cats, birds, rabbits, and lizards. For this study, the focus is on dogs and cats. After filtering through these records, we found that only California, Kentucky, Colorado, Arizona, and Indiana had all of the factors needed for the study. Upon downloading data from the database and receiving data from the animal shelters, the acquired data underwent data integration, data transformation, and data cleaning (as detailed in Fig. 1). After data pre-processing, there are over 113,000 animal records.

### Data cleaning methods

Next, data cleaning methods are utilized to detect discrepancies in the data, such as missing values, erroneous data, and inconsistencies. Data cleaning is an essential step for obtaining unbiased results [35, 36]. In other words, identifying and cleaning erroneous data must be performed before inputting the data into the algorithm as it can significantly impact the output results.

The following is a list of commonly used data cleaning techniques in the literature [11]:

- *Substitution with Median:* Missing or incorrect data are replaced with the median value for that predictor variable.
- *Substitution with a Unique Value:* Erroneous data are replaced with a value that does not fall within the range that the input variables can accept (e.g., a negative number)
- *Discard Variable and Substitute with a Median:* When an input variable has a significant number of missing values, these values are removed from the dataset, and the features that remain with missing or erroneous values are replaced with the median.
- *Discard Variable and Substitute with a Unique Value:* Input variables with a significant number of missing values are removed from the dataset, and the features that remain with missing or erroneous values are coded as − 1.
- *Remove Incomplete Rows Entirely:* Incomplete Rows are removed from the dataset.

### Data preprocessing

Some animal breeds are listed in multiple formats and are changed to maintain uniformity. An example of this is a Russian Blue cat, which is formatted in several ways such as “Russian”, “Russian Blue”, and “RUSSIAN BLUE”. Animals with multiple breeds such as “Shih Tzu/mix” or “Shih Tzu/Yorkshire Terr” are classified as the first breed listed. Other uncommon breeds are classified as “other” for simplicity. Finally, all animal breeds are summarized into three categories (small, medium, or large) using the American Kennel Clubs’ breed size classification [37]. Part of the data cleansing process also includes categorizing multiple colors found throughout the sample size into five distinct color categories (brown, black, blue, white, and multicolor). We classified age into five categories for dogs and cats (puppy or kitten, adolescent, adult, senior, super senior). The puppy or kitten category includes data points 0–1 year, adolescence includes data points 2–3 years old, adulthood includes animals 4–7 years of age, and senior animals are 8–10 years of age. Any animal that is older than ten years are categorized as a super senior, based on the recommendations provided in Wapiti Labs [38].

As mentioned previously, the output variable is the length of stay and is classified as low, medium, high, and very high/euthanization. The length of stay is calculated by taking the difference between the intake date and outcome date. To remove erroneous data entries and special cases, the number of days in the animal shelter is also capped at a year. The “low” category represents animals that are returned (in which case, they are assigned the days in the shelter as 0) or spent less than 8 days before getting adopted. It is important to keep these animals at the shelter so that the owner may find them or they are transferred to their new homes. Animals that stayed in a shelter for 9–42 days and are adopted are categorized as “medium” length of stay. The “high” category is given to animals that stayed in the shelter for 43–365 days. Finally, animals that are euthanized are categorized as “very high”.

After integrating all data points from each animal shelter, the sample size includes 119,691 records. After the evaluation of these data points, 5436 samples are found to have miscellaneous (such as a negative length of stay) or missing values. After applying data cleaning techniques, the final cleaned dataset includes 114,256 data points, with 50,466 cat- and 63,790 dog-records.

### Machine learning algorithms to predict the length of stay

The preprocessed records are then separated into training and testing datasets based on the type of classification algorithm used. Studies have demonstrated the need for testing and comparing machine learning algorithms, as the performance of the models depends on the application. While an algorithm may develop a predictive model that performs well in one application, it may not be the best performing model for another. A comparison between the statistical models is conducted to determine the overall best performing model. In this section, we provide a description as well as the advantages of each classification algorithm that is utilized in this study.

#### Logistic regression

**Fig. 6:**
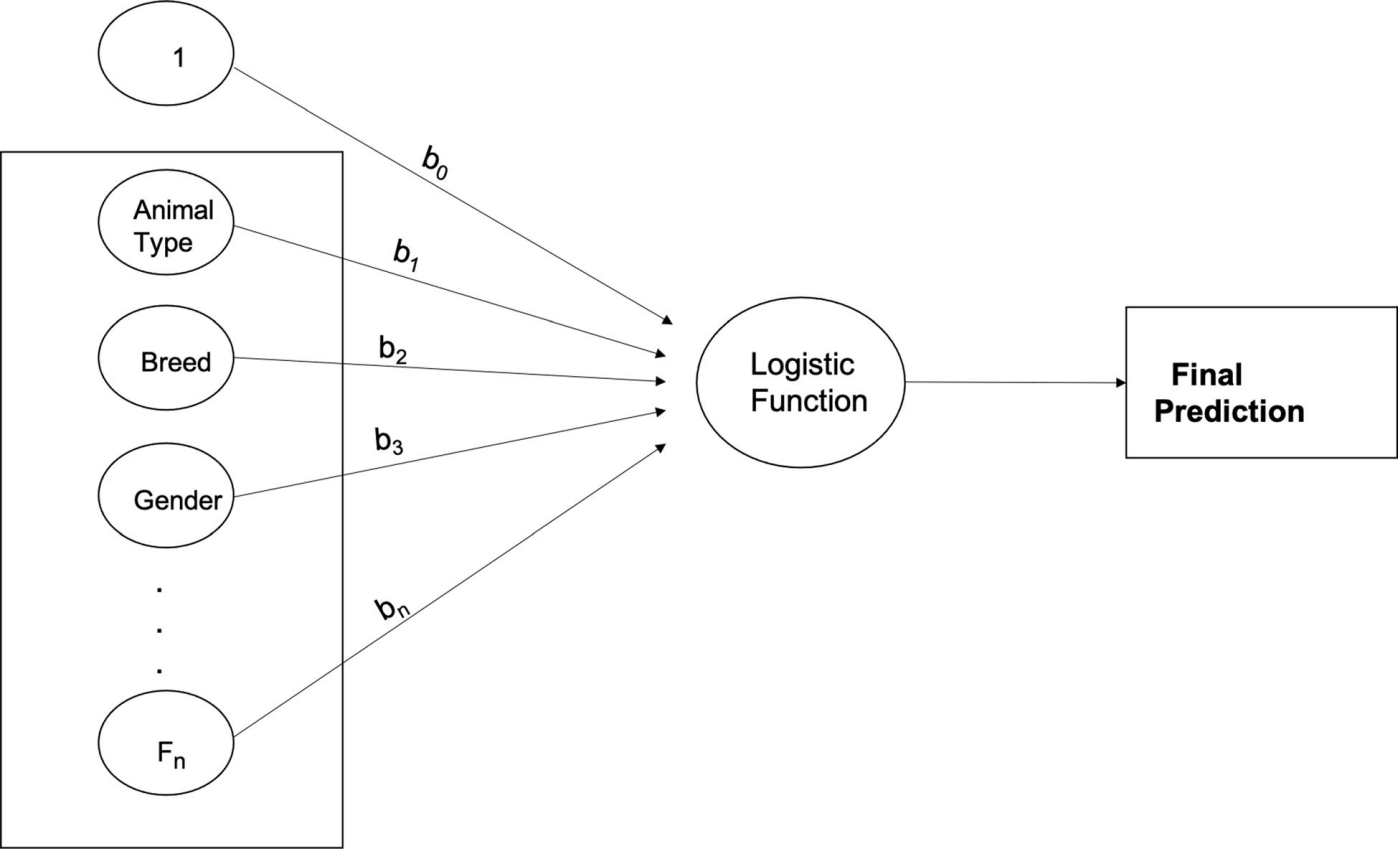
Pictorial Representation of the Logistic Regression Algorithm

Logistic regression (LR) is a machine learning algorithm that is used to understand the probability of the occurrence of an event [39]. It is typically used when the model output variable is binary or categorical (see Fig. 6), unlike linear regression, where the dependent variable is numeric [40]. Logistic regression involves the use of a logistic function, referred to as a “sigmoid function” that takes a real-valued number and maps it into a value between 0 and 1 [41]. The probability that the length of stay of the animal at a specific location will be low, medium, high, or very high, is computed using the input features discussed in Table 4.

The linear predictor function to predict the probability that the animal in record *i* has a low, medium, high, and very high length of stay categories is given by Equations (11) –[3], respectively.

11 $$ f\left( low,i\right)={\beta}_{0,\mathrm{low}}+{\beta}_{\mathrm{type},\mathrm{low}}{x}_{\mathrm{type},\mathrm{i}}+{\beta}_{\mathrm{breed},\mathrm{low}}{x}_{\mathrm{breed},\mathrm{i}}+\dots $$

12 $$ f\left( med,i\right)={\beta}_{0,\mathrm{med}}+{\beta}_{\mathrm{type},\mathrm{med}}{x}_{\mathrm{type},\mathrm{i}}+{\beta}_{\mathrm{breed},\mathrm{med}}{x}_{\mathrm{breed},\mathrm{i}}+\dots $$

13 $$ f\left( high,i\right)={\beta}_{0,\mathrm{high}}+{\beta}_{\mathrm{type},\mathrm{high}}{x}_{\mathrm{type},\mathrm{i}}+{\beta}_{\mathrm{breed},\mathrm{high}}{x}_{\mathrm{breed},\mathrm{i}}+\dots $$

14 $$ f\left(v. high,i\right)={\beta}_{0,\mathrm{v}.\mathrm{high}}+{\beta}_{\mathrm{type},\mathrm{v}.\mathrm{high}}{x}_{\mathrm{type},\mathrm{i}}+{\beta}_{\mathrm{breed},\mathrm{v}.\mathrm{high}}{x}_{\mathrm{breed},\mathrm{i}}+\dots $$

Where *β*_*v*, *l*_ is a set of multinomial logistic regression coefficients for variable *v* of the length of stay category *l*, and *x*_*v*, *i*_ is the input feature *v* corresponding to data observation *i*.

#### Artificial neural network

**Fig. 7:**
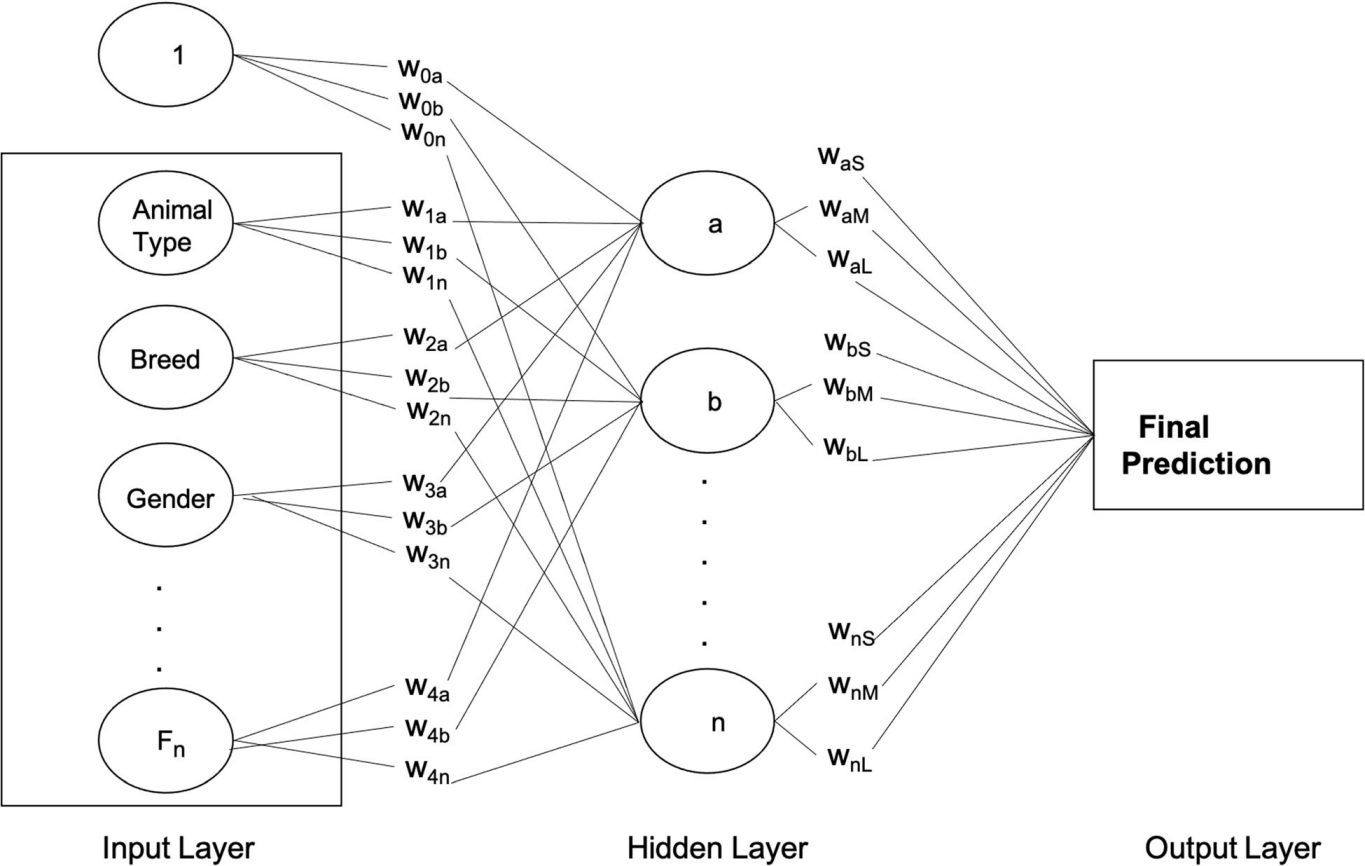
Pictorial Representation of the Artificial Neural Networks

Artificial Neural Network (ANN) algorithms were inspired by the brain’s neuron, which transmits signals to other nerve cells [40, 42]. ANN’s were designed to replicate the way humans learn and were developed to imitate the operational sequence in which the body sends signals in the nervous system [43]. In an ANN, there exists a network structure with directional links connecting multiple nodes or “artificial neurons”. These neurons are information-processing units, and the ties that connect them represent the relationship between each of the connected neurons. Each ANN consists of three layers - the input layer, hidden layer, and the output layer [32, 44]. The input layer is where each of the input variables is fed into the artificial neuron. The neuron will first calculate the sum of multiple inputs from the independent variables. Each of the connecting links (synapses) from these inputs has a characterized weight or strength that has a negative or positive value [32]. When new data is received, the synaptic weight changes, and learning will occur. The hidden layer learns the relationship between the input and output variables, and a threshold value determines whether the artificial neuron will fire or pass the learned information to the output layer, as shown in Fig. 7. Finally, the output layer is where labels are given to the output value, and backpropagation is used to correct any errors.

#### Random Forest

**Fig. 8:**
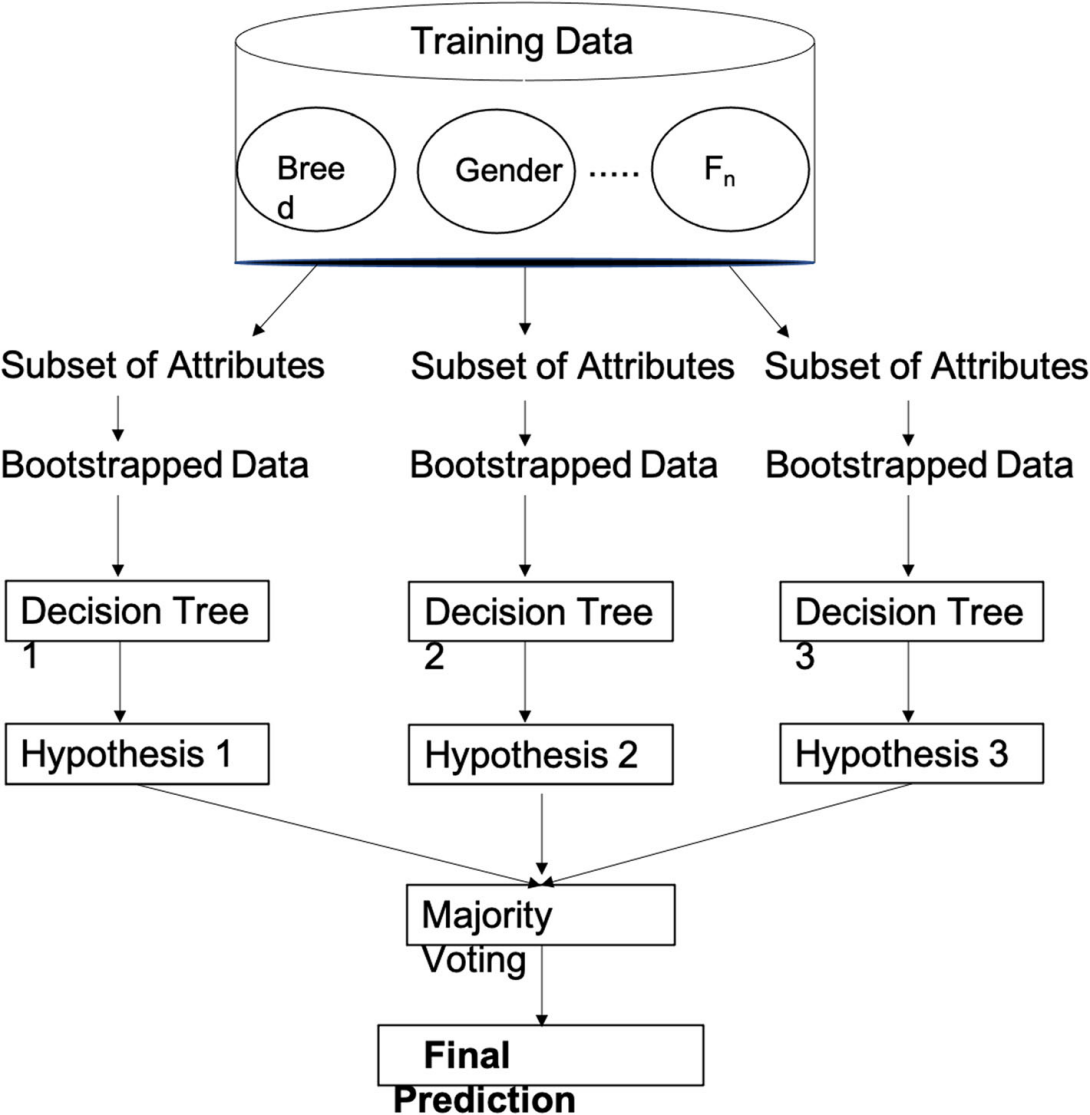
Pictorial Representation of the Random Forest Algorithm

The Random Forest (RF) algorithm is a type of ensemble methodology that combines the results of multiple decision trees to create a new predictive model that is less likely to misclassify new data [30, 45]. Decision Trees have a root node at the top of the tree that consists of the attribute that best classifies the training data. The attribute with the highest information gain (given in Eq. 16) is used to determine the best attribute at each level/node. The root node will be split into more subnodes, which are categorized as a decision node or leaf node. A decision node can be divided into further subnodes, while a leaf node cannot be split further and will provide the final classification or discrete label. RF algorithm uses *mtree* and *ntry* as the two main parameters in developing the multiple parallel decision trees. Mtree specifies how many trees to train in parallel, while ntry defines the number of independent variables or attributes to choose to split each node [30].. The majority voting from all parallel trees gives the final prediction, as given in Fig. 8.

15 $$ \mathrm{Entropy}=\sum \limits_i-{p}_i{\log}_2{p}_i $$

16 $$ \mathrm{Information}\ \mathrm{Gain}=\mathrm{Entropy}\ \left(\mathrm{parent}\right)-\mathrm{Weighted}\ \mathrm{Average}\ \left[\mathrm{Entropy}\ \left(\mathrm{children}\right)\right] $$

#### Gradient boosting

**Fig. 9:**
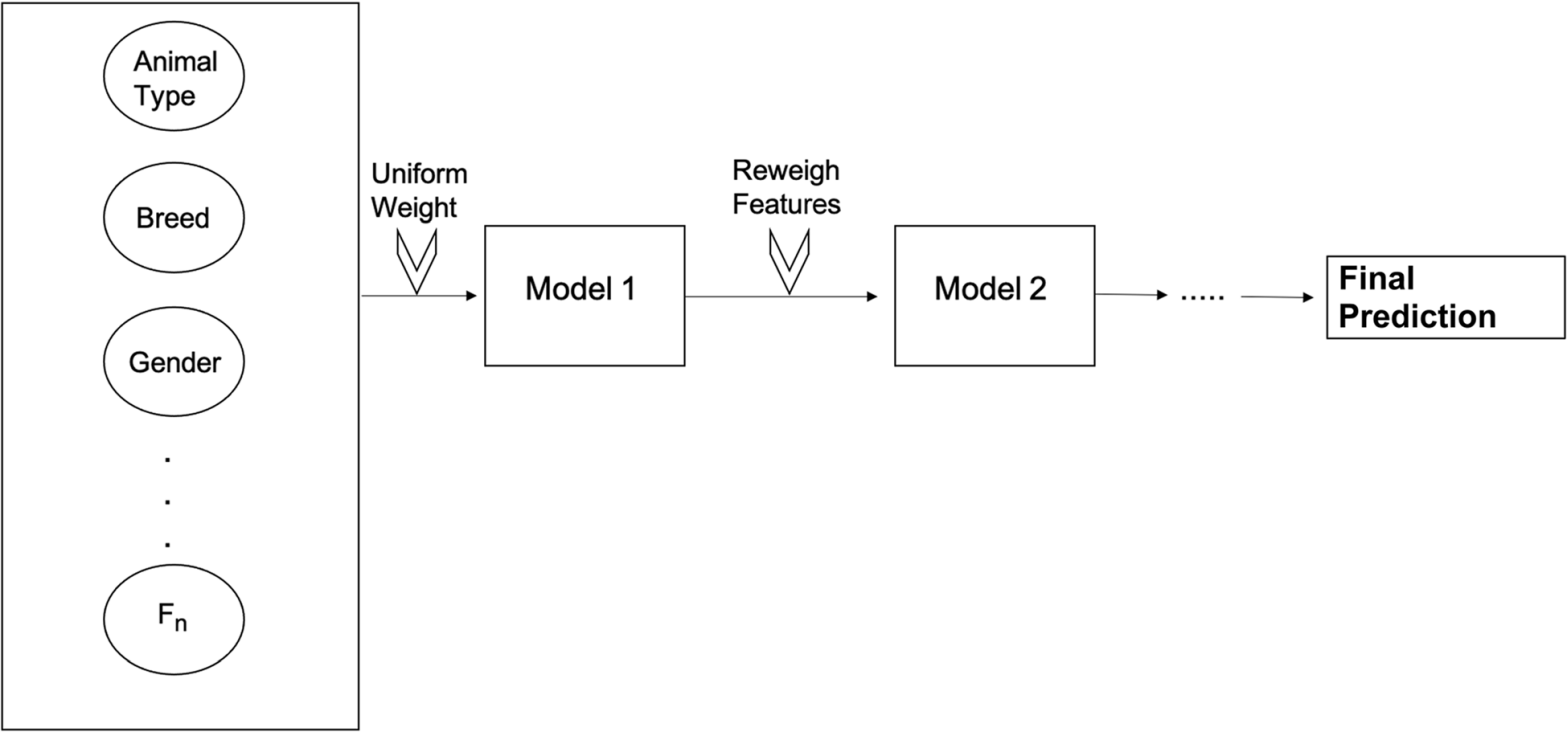
Pictorial Representation of Boosting Algorithm

Boosting is another type of ensemble method that combines the results from multiple predictive algorithms to develop a new model. While the RF approach is built solely on decision trees, boosting algorithms can use various algorithms such as decision trees, logistic regression, and neural networks. The primary goal of boosting algorithms is to convert weak learners into stronger ones by leveraging weighted averages to identify “weak classifiers” [31]. Samples are assigned an initial uniformed weight, and when incorrectly labeled by the algorithm, a penalty of an increase in weight is given [46]. On the other hand, samples that are correctly classified by the algorithm will decrease in weight. This process of re-weighing is done until a weighted vote of weak classifiers is combined into a robust classifier that determines the final labels or classification [46]. For our study, gradient boosting (GB) will be used on decision trees for the given dataset, as illustrated in Fig. 9.

### Machine learning model parameters

The clean animal shelter data is split into two datasets: training and testing data. These records are randomly placed in the two groups to train the algorithms and to test the model developed by the algorithm. 80% of the data is used to train the algorithm, while the other 20% is used to test the predictive model. To avoid overfitting, a tenfold cross-validation procedure is used on the training data. There are no parameters associated with the machine learning of logistic regression algorithms. However, a grid search method is used to tune the parameters of the random forest, gradient boosting, and artificial neural network algorithms. This allows the best parameter in a specific set to be chosen by running an in-depth search by the user during the training period.

The number of trees in the random forest and gradient boosting algorithms is changed from 100 to 1000 in increments of 100. A learning rate of 0.01, 0.05, and 0.10 is used based on the recommendations of previous studies [47]. The minimum observations for the trees’ terminal node are set to vary from 2 to 10 in increments of one, while the splitting of trees varies from 2 to 10 in increments of two. A feed-forward method is used to develop the predictive model using the artificial neural network algorithm. The feed-forward algorithm consists of three layers (input, hidden, output) as well as backpropagation learning. The independent and dependent variables represent the input and output layers. Since the input and output layers are already known, an optimal point is reached for the number of nodes when between 1 and the number of predictors. This means that for our study, the nodes of the hidden layer vary from 1 to 8. The learning rate values used to train the ANN are 0.01, 0.05, and 0.10.

To find the optimal setting for each machine learning algorithm, a thorough search of their corresponding parameter space is performed.

### Performance measures

In this study, we use three performance measures to evaluate the ability of machine learning algorithms in developing the best predictive model for the intended application. The measures considered are precision, F1 score, and sensitivity/recall to determine the best model given the inputted data samples. Table 5 provides a confusion matrix to define the terms used for all possible outcomes.

**Table 5:** Confusion Matrix

		Negative	Positive
**Actual**	**Negative**	True Negative	False Negative
	**Positive**	False Negative	True Negative

Precision evaluates the number of correct, true positive predictions by the algorithm while still considering the incorrectly predicted positive when it should have been negative (Eq. 17). By having high precision, this means that there is a low rate of false positives or type I error. Sensitivity or recall evaluates the number of true positives that are correctly predicted by the algorithm while considering the incorrectly predicted negative when it should have been positive (Eq. 18). Recall is a good tool to use when the focus is on minimizing false negatives (type II error). F1 score (shown in Eq. 19) evaluates both type I and type II errors and assesses the ability of the model to resist false positives and false negatives. This performance metric evaluates the robustness (low number of missed classifications), as well as the number of data points that are classified correctly by the model.

17 $$ Precision=\frac{True\ Positive}{True\ Positive+ False\ Positive\ } $$

18 $$ Sensitivity/ Recall=\frac{True\ Positive}{True\ Positive+ False\ Negative\ } $$

19 $$ F1\  Score=\frac{2\left( True\ Positive\right)}{2\left( True\ Positive\right)+ False\ Positive+ False\ Negative\ } $$

## Data Availability

Most of the datasets used and/or analyzed during the current study were publicly available online as open source data. The data were available in the website details given below: https://data.bloomington.in.gov/dataset https://data.louisvilleky.gov/dataset https://data.sonomacounty.ca.gov/Government We also obtained data from Sun Cities 4 Paws Rescue, Inc., and the Rifle Animal Shelter. No administrative permission was required to access the raw data from these shelters.

## Abbreviations

LR: Logistic Regression
ANN: Artificial Neural Network
RF: Random Forest
GB: Gradient Boosting
GP: Goal Programming
CV: Coefficient of Variation
